# Short-Term Ketogenic Diet Induces a Molecular Response That Is Distinct From Dietary Protein Restriction

**DOI:** 10.3389/fnut.2022.839341

**Published:** 2022-03-30

**Authors:** Krystle C. Kalafut, Sarah J. Mitchell, Michael R. MacArthur, James R. Mitchell

**Affiliations:** ^1^Department of Molecular Metabolism, Harvard T. H. Chan School of Public Health, Boston, MA, United States; ^2^Department of Health Sciences and Technology, Swiss Federal Institute of Technology (ETH) Zürich, Zurich, Switzerland

**Keywords:** ketogenic, dietary restriction, protein restriction, carbohydrate, protein, RNA seq, low protein, carbohydrate restriction

## Abstract

There is increasing interest in utilizing short-term dietary interventions in the contexts of cancer, surgical stress and metabolic disease. These short-term diets may be more feasible than extended interventions and may be designed to complement existing therapies. In particular, the high-fat, low-carbohydrate ketogenic diet (KD), traditionally used to treat epilepsy, has gained popularity as a potential strategy for weight loss and improved metabolic health. In mice, long-term KD improves insulin sensitivity and may extend lifespan and healthspan. Dietary protein restriction (PR) causes increased energy expenditure, weight loss and improved glucose homeostasis. Since KD is inherently a low-protein diet (10% of calories from protein vs. >18% in control diet), here we evaluated the potential for mechanistic overlap between PR and KD *via* activation of a PR response. Mice were fed control, protein-free (PF), or one of four ketogenic diets with varying protein content for 8 days. PF and KD both decreased body weight, fat mass, and liver weights, and reduced fasting glucose and insulin levels, compared to mice fed the control diet. However, PF-fed animals had significantly improved insulin tolerance compared to KD. Furthermore, contrary to the PF-fed mice, mice fed ketogenic diets containing more than 5% of energy from protein did not increase hepatic *Fgf21* or brown adipose *Ucp1* expression. Interestingly, mice fed KD lacking protein demonstrated greater elevations in hepatic *Fgf21* than mice fed a low-fat PF diet. To further elucidate potential mechanistic differences between PF and KD and the interplay between dietary protein and carbohydrate restriction, we conducted RNA-seq analysis on livers from mice fed each of the six diets and identified distinct gene sets which respond to dietary protein content, dietary fat content, and ketogenesis. We conclude that KD with 10% of energy from protein does not induce a protein restriction response, and that the overlapping metabolic benefits of KD and PF diets may occur *via* distinct underlying mechanisms.

## Introduction

The ability of dietary interventions to improve metabolic health and stress resistance has been extensively demonstrated in animal models since the first studies on caloric restriction in the early 1900’s (1–3). Many recent studies have focused on dietary restriction of specific macronutrients, which in some cases has been shown to have similar benefits as total caloric restriction. Both the high-fat, low-carbohydrate ketogenic diet (KD) and protein restricted (PR) diets improve metabolic health and stress resistance in mice (4–7). However, whether there is an overlapping mechanism that underlies the benefits of these diets remains to be determined.

Long-term feeding of both KD and PR diets improves metabolic health in mice. PR diets with a low protein-to-carbohydrate ratio improve measures of cardiometabolic health and glucose tolerance in mice and are associated with lower body weight, and reduced plasma lipids and insulin (8–10). Similarly, KD reduces body weight gain and reduces circulating insulin and glucose levels in mice (11–13) and rats (14, 15). However, studies of short- (3 days) and intermediate-term (3–5 weeks) KD found impaired insulin tolerance and hepatic insulin resistance in mice (12, 16) and rats (14, 17). This contrasts with PR diets which show profound improvements in glucose and lipid homeostasis after just 7 days (18).

Clinically, both KD and PR have a history of long-term use in the treatment of idiopathic epilepsy in adults and children (19–24) and renal failure (25–29), respectively. In addition to effectively treating these conditions, both dietary interventions were observed to improve cardiometabolic parameters in human patients. KD has been shown to cause significant weight loss and reduction in cardiovascular risk factors, including blood pressure, and serum triglycerides and insulin, in non-epileptic humans with obesity (30). PR has been shown to substantially improve glucose and insulin homeostasis in humans with and without diabetes (31–34). In addition, high protein intake is associated with increased overall mortality and mortality from cancer or diabetes in individuals under the age of 65 (35).

Recently, the therapeutic value of short-term or cyclic KD and PR are being investigated for a variety of indications aside from epilepsy and renal insufficiency. Studies in mice have demonstrated that 10 days of KD-feeding can improve the efficacy of PI3K inhibitors in xenograft models (36), and short-term KD was recently shown to impact xenograft tumor growth through alterations in metabolic signaling and nutrient availability (37). Previous pilot studies have demonstrated that KD is safe and feasible in patients with glioblastoma and other advanced cancers (38–40), and there are ongoing pilot studies to evaluate the tolerability of KD in endometrial (NCT03285152) and pancreatic (NCT04631445) cancer patients. Short-term KD has also been proposed to protect against adipose tissue inflammation in mice (41). In addition, a recent report showed that 1-week cycles of KD alternated with a control diet reduces midlife mortality and improves measures of healthspan and memory in mice (42). The fasting mimicking diet (FMD) combines calorie restriction and PR and is being tested as an adjunct to chemotherapy in multiple cancer types (43, 44). Short 4- or 5-day cycles of FMD have also been shown to improve measures of cardiometabolic health in humans (45), and to improve late-life health, included delayed cancer incidence, in mice (46). Preconditioning with short-term PR also effectively protects against ischemic surgical stress in mouse models (7, 47) and a combination of calorie restriction and PR has been shown to be safe in humans prior to surgery (48, 49). Given the recent clinical interest in shorter-term KD and PR diets, it is important to understand the mechanism by which these interventions improve health and stress resistance to fully harness their therapeutic potential.

Both KD and PR are known to induce numerous metabolic adaptations that may contribute to the observed benefits. PR is associated with increases in energy expenditure and thermogenesis, as well as increased circulating fibroblast growth factor 21 (FGF21), a hormone that regulates energy balance by acting on various tissues (50–53). KD is associated with improvements in lipid homeostasis, including upregulation of fatty acid oxidation and fat utilization and decreased lipid synthesis (11, 54). Interestingly, clinical KD diets used in humans are also restricted in protein content, with 5–10% of calories coming from protein compared to 15–20% in normal diets. This raises the question of whether overlapping molecular mechanisms related to reduced dietary protein content underlie the improvements in metabolic health and longevity in response to KD and PR.

The goal of this study was to compare the molecular signatures of mice fed KD to those of mice fed a protein-free (PF) diet. Protein content was titrated between 0–10% in three additional KD diets to evaluate the contribution of dietary protein to metabolic phenotypes associated with KD. After 1 week, PF and KD diets decreased body weight and reduced fasting glucose and insulin levels compared to mice fed the control diet. Insulin tolerance was significantly improved, in PF-fed, but not KD-fed, mice. Contrary to the PF-fed mice, KD-fed mice did not increase markers of the physiological response to protein restriction, including hepatic *Fgf21* or BAT *Ucp1* expression. Liver transcriptomics further illuminated potential shared and independent mechanisms underlying the metabolic adaptations to carbohydrate and protein restriction, including shared alterations in fatty acid metabolism, but distinct changes in amino acid metabolism in PR and nucleotide metabolism in KD. We conclude that KD does not induce a robust protein restriction response, and that the overlapping metabolic benefits of KD and PF diets may be explained by distinct underlying mechanisms.

## Materials and Methods

### Mice

16-week-old male B6D2/F1 hybrid mice were purchased from Jackson Labs (strain no. 100006). Mice were acclimated to the Harvard T. H. Chan School of Public Health mouse facility for 2 weeks prior to experiments and were fed a standard chow diet during this acclimation period (Purina, 5053-PicoLab Rodent Diet 20). Mice were allowed *ad libitum* access to food and water unless otherwise noted. Mouse numbers were *n* = 4 per diet group for the GTT, ITT, and insulin injection experiments, and a separate cohort of *n* = 6 per diet group for all other analyses, with 2 mice co-housed per cage. Mice were maintained at 22°C with 12-h light-dark cycles and 30–50% relative humidity. Mice were housed in a Specific Pathogen Free (SPF) facility, as determined by screening of sentinel animals at monthly intervals. Mice were fasted for 6 h prior to sacrifice and tissue collection. All procedures were approved by the Harvard Institutional Animal Care and Use Committee.

### Diets

Semi-purified diets were prepared using combinations of soluble protein-free base mixtures D12450Spx or D100070801Lpx (Research Diets, Supplementary Tables 1, 2), cocoa butter, casein, cystine and sucrose (Supplementary Figure 1B). Mixtures were combined with an equal mass of 2% agar in water to form a solid gel diet. Diets had macronutrient compositions and energy densities as listed in Supplementary Figure 1A. Food intake was measured and food replenished as needed daily at approximately ZT10, or 10 h into the mouse light cycle.

### Body Composition

Body mass was determined by daily measurement at approximately ZT10. Lean and fat mass were measured in awake mice using an EchoMRI analyzer system.

### Serum Measurements

Mice were fasted for 6 h (ZT02-ZT08) prior to measurements and tail vein blood collection. Glucose was measured using a Clarity BG1000 handheld glucometer. Serum insulin was measured by ELISA following manufacturer protocol (Crystal Chem, #90080). Ketones were measured using a Nova Max Plus Glucose/Ketone handheld meter with Nova Max Ketone Strips. Serum FGF21 was measured by ELISA following manufacturer protocol (R&D Systems, #MF2100).

### Glucose and Insulin Tolerance Tests

Metabolic assessments were performed in a separate cohort of mice fed either CTL, PF, or KD10P diets (*n* = 4 per diet). An oral glucose tolerance test (OGTT) was performed on day 6 and an insulin tolerance test (ITT) was performed on day 8. For OGTT, mice were fasted for 6 h and then 30% D-glucose (Sigma Aldrich) in sterile water was administered by oral gavage at 2 g/kg. Blood glucose was measured from a small tail nick prior to (time 0) and at 15-, 30-, 60-, and 120-min post-gavage using a handheld glucometer (Clarity BG1000). Blood was collected *via* tail vein at all timepoints, and serum was generated for insulin measurement.

For ITT, mice were fasted for 4 h then injected intraperitoneally with 1.5 IU/kg. Blood glucose was measured from a small tail nick prior to (time 0) and at 15-, 30-, 60-, and 120-min post-injection as above.

### Insulin Signaling

Mice fed CTL, PF, or KD10P diets for 10 days (*n* = 4 per group) were fasted for 6 h and then injected with 1.5 IU/kg insulin or vehicle for 20 min. Liver tissue was collected and flash frozen prior to analysis.

### Immunoblotting

Flash-frozen liver tissue was homogenized and lysed in RIPA buffer (50 mM Tris–Cl pH 7.4, 150 mM NaCl, 1% IGEPAL, 0.5% sodium deoxycholic acid, 0.1% SDS, 1 mM EDTA, 10 mM NaF, 10 mM sodium pyrophosphate, 1 mM β-glycerophosphate, and 1 mM sodium orthovanadate, Sigma protease inhibitor P8340). Protein quantification was performed using a BCA assay kit (Thermo Scientific) and equal amounts of protein were separated by SDS-PAGE, transferred to nitrocellulose membranes, and immunoblotted with indicated primary antibodies. Primary antibodies: p-IRS1 (CST, 2381), IRS (CST, 2382), p-FoxO1 (CST, 9464), FoxO1 (CST, 9462), p-Akt (CST, 4060), Akt (CST, 4691), p-rpS6 S235/36 (CST, 2211), rpS6 (CST, 2217), and Actin (CST, 4967). Secondary antibodies: anti-rabbit IgG, HRP-linked (CST, 7074), anti-mouse IgG, HRP-linked (CST, 7076). Immunoblots were developed by ECL (West Pico or Femto, Thermo Scientific).

### Real-Time Quantitative Polymerase Chain Reaction

RNA was isolated from flash-frozen liver or brown adipose tissue by homogenization in TRIzol Reagent (Thermo Fisher) followed by chloroform extraction and isopropanol precipitation. The concentration of RNA was determined using a Nanodrop Spectrophotometer. RNA (1 μg) was reverse transcribed using the Advanced cDNA Synthesis Kit (Bio-Rad). qPCR was performed using Power Up SYBR green (Bio-Rad) with duplicate technical replicates using the QuantStudio 5 Real-Time PCR system. ΔΔCt values were normalized to actin and relative expression was plotted. Primer sequences were: Asns forward: GCAGTGTCTGAGTGCGATGAA, Asns reverse: TCTTATCGG CTGCATTCCAAAC, Fgf21 forward: CTGCTGGGGGTCTA CCAAG, Fgf21 reverse: CTGCGCCTACCACTGTTCC, Ucp1 forward: AGGCTTCCAGTACCATTAGGT, Ucp1 reverse: CTGAGTGAGGCAAAGCTGATTT.

### RNA-Seq

Livers were collected from euthanized mice and immediately flash frozen in liquid nitrogen and stored at −80°C until analysis. Livers were homogenized using a handheld homogenizer and RNA was extracted using a Qiagen RNeasy Plus Mini Kit (Qiagen #74134). The concentration and purity of RNA was determined using a Nanodrop Spectrophotometer and confirmed using an Agilent 2100 Bioanalyzer. Libraries were prepared using the Illumina TruSeq Stranded Total RNA Sample Preparation protocol. RNA was sequenced on an Illumina NovaSeq 6000 with 20 million paired end reads (150 bp length) per sample.

Reads were aligned to the mouse GRCm38.p6 assembly using the align function and annotated using the featureCounts function from the Rsubread package (version 2.3.7). Differential expression analysis was performed using the edgeR (3.30.3) and limma (3.44.3) packages. Gene symbols were mapped to Entrez IDs using the mapIds function from the AnnotationDbi package (1.51.1). Cluster analysis was performed using the degPatterns function from the DEGreport (1.24.1) package. Normalization was performed using the trimmed mean of *M*-values method as implemented in the calcNormFactors from edgeR. Data were modeled and differential expression was determined using the limma voom pipeline to generate linear models with empirical Bayes moderation. Differential expression was determined using a Benjamini–Hochberg adjusted *p*-value less than 0.05. Once differentially expressed genes or gene clusters were determined, gene set enrichment analysis was determined using the enrichKEGG function from the clusterProfiler package (3.16.0) (55). Weighted gene correlation network analysis was performed using the WGCNA package (1.70-3) (56). Transcription factor (TF) binding analyses were performed using CiiiDER (57).

### Statistics

Statistical analyses were performed in Prism (version 8, GraphPad Software) and mean values were plotted with error bars representing standard deviations. One-way ANOVAs with a significant F-statistic were followed by either Tukey’s *post hoc* test or Dunnett’s *post hoc* test. Transcriptomic data were analyzed using R (version 4.0.2) and multiple comparisons in transcriptomic data were corrected using the Benjamini-Hochberg false detection rate correction.

## Results

### Short-Term Dietary Protein or Carbohydrate Restriction Reduces Body Weight and Adiposity in Mice

**FIGURE 1:**
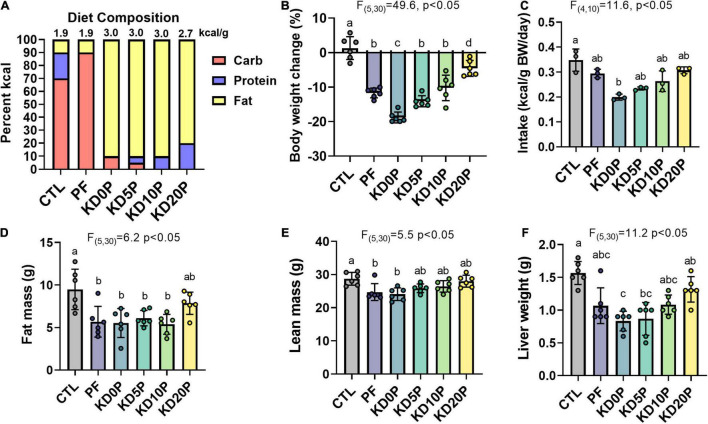
Short-term dietary protein or carbohydrate restriction alters body composition. Male B6D2F1 mice were fed one of six diets for 8 days (*n* = 6 per diet) with varying macronutrient compositions as depicted in **(A)**. The energy density of each diet is listed above the associated bar. **(B)** Changes in body weight after 8 days of diet shown as the percent of the initial weight. **(C)** Average energy intake across 8 days of diet feeding. Intake was normalized to the summed body weight of 2 mice co-housed per cage and presented per cage. Fat mass **(D)** and lean mass **(E)** quantified by EchoMRI at the end of the diet period. **(F)** Liver weights at sacrifice after 6 h fasting period. Statistical analysis was performed by one-way ANOVA with Tukey’s multiple comparison test **(A–D)**. Different letters indicate significant differences (*p* < 0.05), with common letters indicating no significant difference between groups. Values are represented as mean ± SD.

In order to evaluate the contribution of protein restriction to the metabolic and molecular adaptations elicited by KD, mice were fed six distinct diets: Low-fat control containing 20% of energy from protein (CTL) or 0% of energy from protein (PF), classical KD containing no carbohydrate and 10% energy from protein (KD10P), modified KD for which half or all protein content was replaced with carbohydrate (KD5P or KD0P), or KD with protein content normalized to the control diet (KD20P) (Figure 1A and Supplementary Figures 1A,B).

Sixteen-week-old male B6D2F1 mice were fed the above diets for 8 days. Body weight and food consumption were tracked across the diet period (Supplementary Figures 1C,D). All experimental diets resulted in significant weight loss compared to mice fed the CTL diet, with KD0P resulting in the greatest magnitude of weight loss (Figure 1B). A similar degree of weight loss was observed following PF and KD10P diets. All diets tended to have a lower energy intake vs. control, but only KD0P had a statistically significant decrease compared to control (Figure 1C). Weight loss was primarily attributed to significant reductions in fat mass, which was observed in all diet groups, except KD20P (Figure 1D). However, significant reductions in lean mass were also observed in PF and KD0P groups (Figure 1E). Liver weight was also reduced following all experimental diets, with KD0P and KD5P groups significantly different from CTL mice (Figure 1F).

### Protein-Free Diet Improves Insulin Tolerance Compared to Ketogenic Diet Following Short-Term Feeding

**FIGURE 2:**
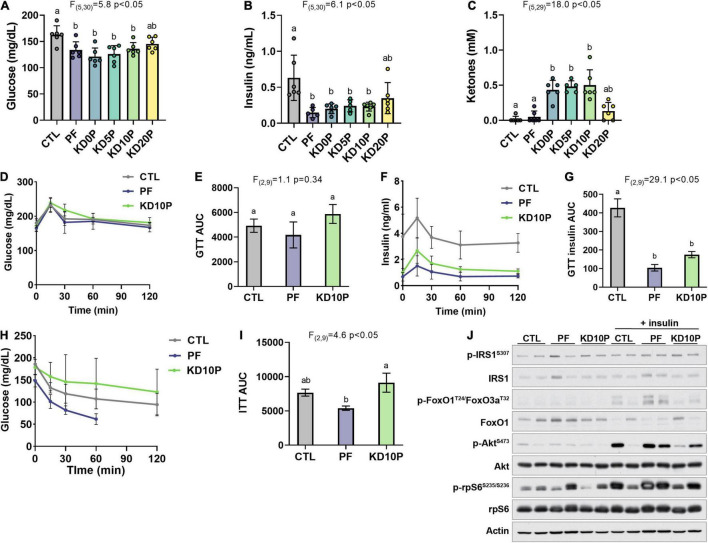
Protein-free, but not ketogenic, diet improves insulin sensitivity following short-term feeding. After 8 days of diet feeding, mice were fasted for 6 h prior to sacrifice (*n* = 6 per diet). Blood was collected and blood glucose **(A)** and ketones **(C)** were measured with handheld meters. Serum insulin was measured by ELISA **(B)**. **(D)** Blood glucose levels during an oral glucose tolerance test (OGTT) in a separate cohort of 6-h fasted mice fed experimental diets for 5 days (*n* = 4 per diet) and **(E)** corresponding area under the curve (AUC). **(F)** Serum insulin measurements collected during the OGTT and **(G)** corresponding AUC. **(H)** Blood glucose levels during an insulin tolerance test in 4-h fasted mice fed experimental diets for 7 days (*n* = 4 per diet) and **(I)** corresponding AUC. Measurements were halted after 60 min in PF-fed mice due to severe hypoglycemia. **(J)** Mice fed experimental diets for 10 days were fasted for 6 h and then injected intraperitoneally with vehicle or insulin and sacrificed after 20 min. Immunoblots show insulin/Akt/mTORC1 signaling in liver lysates (*n* = 2 per diet per treatment). Statistical analysis was performed by one-way ANOVA with Tukey’s multiple comparison test. Different letters indicate significant differences (*p* < 0.05) between groups, with common letters indicating no significant difference. Values are represented as mean ± SD.

To evaluate the metabolic effects of short-term protein or carbohydrate restriction, markers of systemic glucose homeostasis were assessed. Mice fed PF and KD diets, except for KD20P, had significantly lower fasting blood glucose and serum insulin after 1 week (Figures 2A,B). KDs with 0–10% of energy from protein resulted in elevated fasting ketone levels, which was blunted by the addition of protein to 20% of energy (KD20P) (Figure 2C). Increased ketone levels were not observed in mice fed PF.

Glucose and insulin tolerance were further assessed in a separate cohort of mice fed CTL, PF, or KD10P diets (n = 4 per diet). Neither PF nor KD10P diets significantly affected glucose tolerance (Figures 2D,E and Supplementary Figures 2A,B). Serum insulin concentrations during the GTT were significantly lower in both PF- and KD-fed mice (Figures 2F,G), suggesting that mice fed PF and KD10P require less insulin to clear exogenous glucose. However, PF-fed mice demonstrated significantly greater insulin tolerance compared to KD mice (Figures 2H,I and Supplementary Figures 2C,D).

To evaluate whether PF or KD10P diets enhanced the hepatic response to insulin, insulin/Akt signaling was assessed in liver tissue following intraperitoneal insulin injection (*n* = 2 vehicle, 2 insulin per diet). Liver Akt activation, indicated by phosphorylation of Akt and its target FoxO1, was induced in response to insulin in all mice regardless of diet. While conclusions are limited by the small sample size of this experiment, insulin-induced Akt activation appeared to be enhanced in PF compared to KD10P mice (Figure 2J and Supplementary Figures 2E,F).

### Protein-Free, but Not Ketogenic, Diets Induce Molecular Markers of Dietary Protein Restriction

**FIGURE 3:**
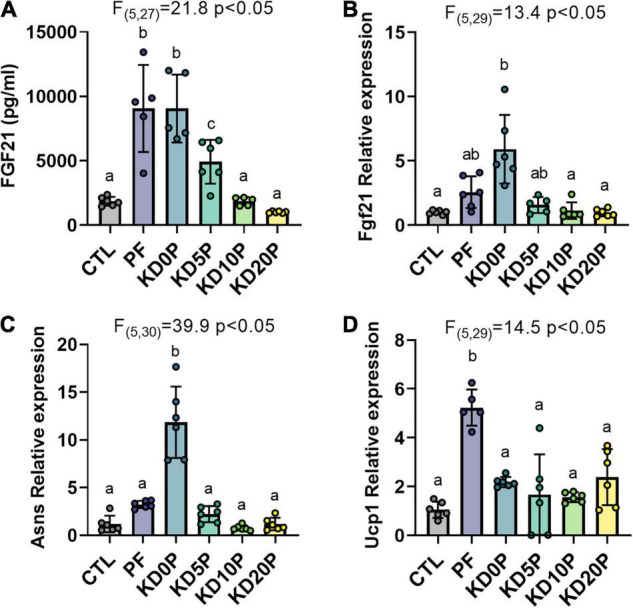
Protein-free, but not ketogenic, diets induce molecular markers of dietary protein restriction. **(A)** Serum FGF21 was measured in 6-h fasted mice fed experimental diets for 8 days. Liver gene expression of *Fgf21* **(B)** and *Asns* **(C)** and brown adipose tissue expression of *Ucp1* **(D)** were measured by RT-qPCR and normalized to actin expression. Statistical analysis was performed by one-way ANOVA with Tukey’s multiple comparison test. Different letters indicate significant differences (*p* < 0.05) between groups, with common letters indicating no significant difference. Values are represented as mean ± SD.

Next, we aimed to determine whether ketogenic diets induce established markers of protein restriction, including serum FGF21 levels, hepatic *Fgf21* and *Asns* mRNA expression and brown adipose tissue (BAT) *Ucp1* mRNA expression. Serum FGF21 was increased ∼5-fold in mice fed either protein-free diets, PF or KD0P, compared to CTL mice (Figure 3A). Serum FGF21 was also significantly increased in mice fed KD5P, but to a lesser extent, and was not increased in mice fed either KD10P or KD20P, compared to CTL mice.

The adaptation to dietary protein restriction includes upregulation of activating transcription factor 4 (ATF4) gene targets *Fgf21* and *Asns* in the liver, as well as upregulation of *Ucp1* expression in BAT. Next, we compared the induction of these gene signatures by PF and KD diets. As expected, liver *Fgf21* and *Asns* expression was elevated in mice fed PF (Figures 3B,C). However, neither *Fgf21* nor *Asns* expression was increased in the liver of mice fed KDs containing at least 5% protein (Figures 3B,C). In agreement with previous reports, BAT *Ucp1* expression was significantly elevated in mice fed PF compared to those fed CTL (∼5-fold, Figure 3D). While BAT *Ucp1* expression was slightly increased in mice fed either of the KD diets compared to those fed the CTL diet, none of these differences were significant. Interestingly, although mice fed the protein-free KD, KD0P, demonstrated significantly greater elevations in hepatic *Fgf21* (∼2-fold difference) and *Asns* expression (∼4-fold difference) compared to mice fed the carbohydrate-rich PF diet, they showed only equivalent or less circulating FGF21 and BAT *Ucp1* expression (Figures 3A–D).

### Protein-Free and Ketogenic Diets Produce Distinct Hepatic Transcriptomic Signatures

**FIGURE 4:**
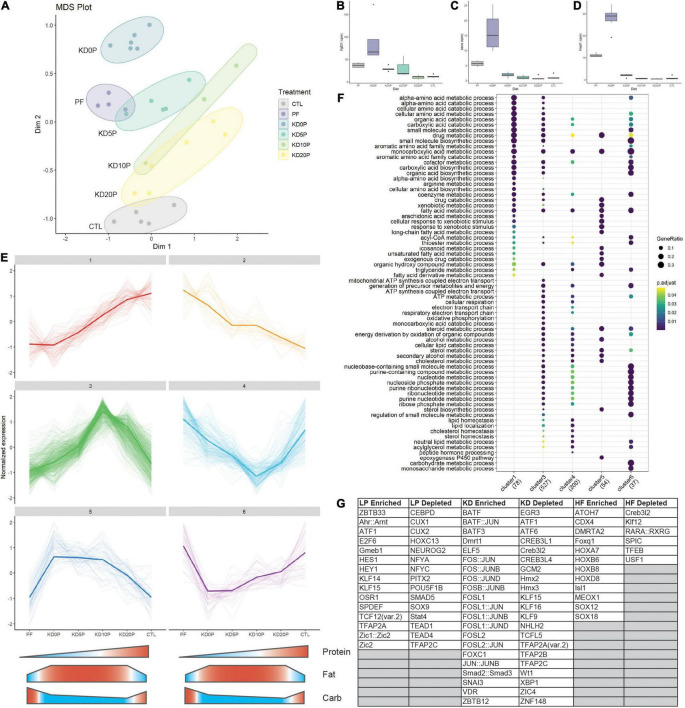
Protein-free and ketogenic diets produce distinct hepatic transcriptomic signatures. **(A)** Multidimensional scaling (MDS) plot across all diet groups/samples. **(B)** Expression of amino acid responsive genes Fgf21, **(C)** Asns and **(D)** Psat1 presented at counts per million (cpm). **(E)** Cluster analysis of the top 10% of most variable genes in the dataset. Clusters 1 and 2 represent protein-responsive genes. Clusters 3 and 4 represent genes responding to classic ketogenic diet. Clusters 5 and 6 include genes responsive to dietary fat content. **(F)** Pathway over-representation analysis of genes from the clusters identified in **(E)**. **(G)** Enriched and depleted transcription factor (TF) binding motifs in the clusters identified in E. LP enriched includes TF binding motifs which are significantly depleted in cluster 1 and significantly enriched in cluster 2. The same method was used for KD in clusters 3 and 4 and high fat (HF) in clusters 5 and 6.

To further explore changes in hepatic gene expression as a function of diet, we performed RNA sequencing. Data reduction by multidimensional scaling showed distinct clustering of samples by group with KD20P showing the least divergence from CTL and KD0P showing the greatest divergence (Figure 4A). Key genes in the amino acid restriction response showed a greater relative increase in KD0P vs. PF, and no response in KD10P, including *Fgf21*, *Asns*, and *Psat1* (Figures 4B–D). Interestingly, although *Atf4* expression was similarly elevated in KD0P compared to PF livers, it also tended to increase across the other KD groups (Supplementary Figure 3A), suggesting it may not be a key driver of this response. When weighted gene correlation network analysis (WGCNA) was performed, a single module containing these three genes (*Fgf21*, *Psat1*, and *Asns*) was identified. This module contained 51 genes which exhibited similar behavior across groups including a proportionally greater increase in KD0P vs. PF (Supplementary Figure 3B). Several other genes characteristic of the amino acid stress response were identified in this module including tRNA synthetases (*Yars*, *Cars*, *Mars*, *Farsa*, and *Rars*), *Pck2* and *Mthfd2*. When transcription factor (TF) binding site analysis was performed, binding motifs for Ventx, Thap11, Atf4, and Sox21 were the top enriched hits compared to a background list of 1000 random genes (Supplementary Figure 3C). Binding motifs for multiple FOX family TFs were significantly depleted in this module, including Foxo4, Foxo6, and Foxl1 (Supplementary Figure 3C).

To better understand how gene networks change in response to the experimental diets, unsupervised clustering of the most variable 10% of genes in the dataset was performed. Six major clusters were identified from this analysis (Figure 4E). Clusters 1 and 2 responded to dietary protein content by linearly increasing or decreasing, respectively. Clusters 3 and 4 were either increased or decreased, respectively by the classic ketogenic diet (KD10P). Clusters 5 and 6 were comprised of genes which increased or decreased, respectively as a function of increased dietary fat content, largely independent of ketosis.

Gene set overrepresentation analysis showed that the genes linearly responding to protein content were largely involved in amino acid metabolic processes, fatty acid metabolism and CoA metabolic processes (Figure 4F). Cluster 3 (increasing with classic KD) was enriched for genes involved in oxidative phosphorylation, lipid catabolism and nucleotide metabolic processes, which were not observed in the protein responsive clusters. There was some overlap between clusters 1 and 3 regarding amino acid catabolic processes. The pathways increased in response to increased dietary fat content (cluster 5) were primarily involved in lipid metabolism and CYP regulation. Interestingly, pathways enriched in genes that decreased in response to dietary fat tended to overlap with those that were increased in response to classic KD, particularly those involving nucleotide metabolism.

To investigate TFs that may be regulating these differential responses to diet, binding site motif analysis was performed across the clusters. To determine TFs that may be enriched in response to low protein, the overlap of TFs significantly depleted in cluster 1 and significantly enriched in cluster 2 was taken. A similar approach was taken to identify TFs enriched by KD using clusters 3 and 4, and TFs enriched by high fat using clusters 5 and 6. In the low protein condition, motifs for Atf1, Ahr:Arnt and E2F6 binding were among the most enriched (Figure 4G and Supplementary Figures 3D,E). In the KD condition, binding sites for FOS and JUN family proteins were highly enriched including Fos, Fosb, Fosl1, and Fosl2 (Figure 4G and Supplementary Figures 3F,G). In the high fat condition, binding motifs for HOX and SOX family proteins were significantly enriched, independent of ketogenesis (Figure 4G and Supplementary Figures 4H,I).

## Discussion

This study utilized six distinct diets to dissect the influence of protein content on the short-term metabolic adaptations to the high-fat, low-carbohydrate ketogenic diet. After 8 days, the high-carbohydrate protein-free diet (PF) and three ketogenic diets (KDs) with 0, 5, or 10% energy from protein resulted in weight loss, which was primarily attributed to loss of fat mass, and reduced fasting blood glucose and insulin levels. Despite similar physiological effects of KD and PF diets, the diets differed with respect to some metabolic and molecular markers. Mice fed PF demonstrated significantly greater insulin tolerance than mice fed KD10P. With the exception of the protein-free KD (KD0P), KD diets did not lead to elevations in serum or tissue markers of protein restriction, including circulating FGF21, hepatic *Fgf21* and *Asns* and BAT *Ucp1* expression. Furthermore, PF and classic KD diets elicited distinct changes in global gene expression. These data indicate that KD does not induce a molecular response similar to protein restriction and that PF and KD diets likely impact health and metabolism *via* unique underlying mechanisms.

Defining the effects of KD on metabolic health has been complicated by discrepancies in diet composition across studies, particularly in studies which use “control chow” diets. KD diets in published rodent studies contain between 4.5–20% protein with feeding durations from 3 days to 14 months. The present study helps clarify discrepancies by using standardized semi-purified diets that control for micronutrient composition and span the range of protein content found in published KD diets. In addition to KD diet composition, published studies differ with respect to control diet formulation. For studies that utilize a semi-purified ketogenic diet, the control diet should also be semi-purified in order to ensure that differences in micronutrient content, nutrient source or other non-nutritive components do not account for any of the phenotypic observations attributed to macronutrients. The diets used in the present study were prepared from the same semi-purified ingredients in order to evaluate the precise contribution of individual macronutrients.

Dietary fat source is another potential source of discrepancy across rodent KD studies. All KDs in this study contained cocoa butter as a fat source, while many published studies utilized the commercial KD from Bio-Serv (F-3666), which contains lard, butter, and corn oil. Cocoa butter differs from these sources in that it is plant-based, while the fat in F-3666 is primarily animal-based. In addition, these fat sources have different fatty acid compositions. Cocoa butter contains predominantly saturated fatty acids (about 60%), while the commercial diet contains 42.5% saturated and 57.5% unsaturated fatty acids. It has been shown that dietary fat source can impact the degree of lifespan extension by caloric restriction (58). Furthermore, mice fed a high fat diet containing lard had elevated insulin and HOMA-IR measurement of insulin resistance after 20 weeks compared to mice fed a plant-based high fat diet (soy and cotton oil) or a standard diet (59). In addition, a study in rats found that insulin resistance in diet-induced obesity was worse in response to high fat diets based on lard or olive oil, which are high in saturated and/or monounsaturated fatty acids, compared to those based on coconut fat or fish oil (60). Thus, although it is clear that dietary fat composition can modify many metabolic health parameters, more work is required to understand the effect of dietary fat composition in the context of KD.

It is also important to note that some effects of KD may be specific to the duration of feeding. Our finding that mice fed KD for 8 days do not have improved insulin tolerance, despite reduced blood glucose and insulin levels, is aligned with other studies that found no change or worsened insulin tolerance after 3 days (16) or 5–7 weeks KD (12, 61) in mice. However, Douris et al. found improved insulin sensitivity in mice fed KD for 8 weeks (13), along with many others reporting benefits beyond 8 weeks, indicating that beneficial adaptations occur in response to longer-term KD feeding. Animals must adapt to carbohydrate restriction by improving the efficiency of fat oxidation in order to support prolonged ketosis and to sustain energy homeostasis (62). There is no consensus regarding the time it takes for rodents or humans to fully adapt to carbohydrate restriction, which has important implications for clinical trials using shorter-term interventions for example NCT03285152 and NCT04631445 in endometrial and pancreatic cancer. This study utilized short-term feeding to compare the physiological and molecular responses of KD vs. PF diets within a time window that may be more relevant for clinical applications like cancer adjuvant therapy.

The present study found no elevations in serum FGF21 and no induction of hepatic *Fgf21* expression in mice fed the classic KD containing 10% protein. Published studies utilizing KD with 5% protein energy report increased plasma or hepatic FGF21 after 4–5 weeks (12, 61). Badman et al. show that FGF21 is required for lipid-based adaptations to KD with 5% protein (63), while Stemmer et al. show that FGF21 is not required for improved glucose homeostasis or ketosis upon KD with 5% or 13% protein (64). Multiple studies demonstrate that supplementing a 5% protein KD up to 10% or 13% protein with fat or carb substitution abolishes FGF21 induction without substantially affecting ketogenesis (64, 65). This supports the notion that FGF21 induction and downstream thermogenesis is a response to low protein levels, and not ketogenesis *per se*. By carefully titrating the levels of protein in KD we further demonstrate that circulating FGF21 is induced in response to decreasing dietary protein, and not carbohydrate, content. This result is consistent with a published report demonstrating that low protein, high carbohydrate diets are associated with maximal FGF21 induction (66) and demonstrates that KD may act independently of dietary protein restriction to elicit metabolic or other health benefits. The classic clinical ketogenic protocol is a 4:1 ratio of fat to protein/carb, resulting in ∼90% of energy from fat and 10% from protein/carb. This is in contrast with the most commonly used rodent KD, the Bio-Serv F3666, which contains 5% of energy from protein. Thus, we propose that future translational studies on KD utilize diets containing at least 10% protein to ensure that observations are due to carbohydrate, rather than protein restriction.

FGF21 signaling in the central nervous system (CNS) contributes to increased food intake during low-protein diet (5% protein) feeding, a phenomenon known as protein leverage (67). However, FGF21 signaling is not sufficient to overcome reduced food intake and body weight in response to very-low-protein diets (1% protein). This decrease in intake is at least partially attributed to reduced hypothalamic mTOR signaling (68, 69). Interestingly, increased BAT *Ucp1* expression requires intact FGF21 signaling in the brain, but not in the adipose tissue (67). In our study, mice fed the high-fat, protein-free diet (KD0P) had equal serum FGF21 and hepatic *Fgf21* expression levels vs. high-carbohydrate PF-fed mice. However, the KD0P group had significantly reduced energy intake and did not have elevated BAT *Ucp1* expression compared to mice fed CTL and PF diets. Given that brain FGF21 signaling promotes energy intake and is required for *Ucp1* induction, it is possible that these characteristics of mice fed the high-fat KD0P are a result of FGF21 resistance, which has been associated with high-fat diet-induced obesity in mice (70). This may also explain why markers of protein restriction are induced in response to KD0P in the liver, but not BAT.

Consistent with this observed difference, on the transcriptional level PF and KD drove divergent gene expression programs. First, the overall transcriptional response to PF diet was amplified by substituting carbohydrate for fat (PF vs. KD0P, Figure 4A). The high fat background drove higher expression of multiple genes involved in amino acid synthesis including *Psat1*, *Phgdh*, and *Asns*, as well as the amino acid responsive transcription factor *Atf4*. WGCNA analysis identified a module containing these amino acid responsive genes which were further increased by high fat background. Atf4 was the third-most enriched TF-binding motif among these genes. However, *Atf4* transcript level also increased in KDs where these amino acid-related targets did not increase. This leaves an open question about the role of ATF4 in driving this differential expression in response to KD0P vs. PF.

Clustering analysis reveal expression modules which were responsive to protein, to classic KD (KD10P) and to fat content independent of protein or ketogenesis. Interestingly, the largest number of genes were positive responders to classic KD, with over 500 genes clustering in this pattern. The protein responsive clusters were primarily enriched for genes involved in amino acid metabolism and fatty acid metabolism, with increased expression of genes involved in non-essential amino acid synthesis and lipid degradation pathways. Among the most enriched pathways in the classic KD responsive gene set was oxidative phosphorylation, with expression of many mitochondrial genes, such as *Ndufa10*, *Sdhb*, and *Sdhc* increased in response to KD10P. Interestingly, these genes tended to be suppressed by reduced protein, even in the high fat background. Similarly, nucleotide metabolic processes were also enriched in response to classic KD (cluster 3). These pathways show some overlap with mitochondrial-related pathways and include genes like *Mpc2*, *Upp2*, and *Pmvk*. Many of these nucleotide-related processes tended to show an overall suppression by high fat diet, suggesting that these increases are specific to the classic ketogenic diet, and not just due to high fat content. TF analyses showed enrichment of Atf and Hif signaling in low protein groups, with Atf1, Atf7, and Hif/Arnt showing strong enrichments. In the KD clusters, binding motifs for Fos/Jun were robustly enriched. These differential TF signatures support the divergent expression profiles seen in the gene expression analysis.

While this study clarifies that protein restriction shows relatively little overlap with KD, multiple outstanding questions remain. In particular, it will be important to understand how the molecular and metabolic adaptations to KD differ across feeding duration and how short vs. long-term adaptations are connected to stress resistance, weight loss and glucose homeostasis. In addition, it is unknown how ketosis or other KD-induced alterations impart the associated physiological changes. The transcriptomics data presented here provide novel insight into potential underlying mechanisms of KD and can serve as a starting point for future mechanistic studies. In conclusion, KD and protein-restricted diets produce similar physiological outcomes, though through distinct mechanisms, and more work is needed to define how short-term KD may be utilized therapeutically.

## Data Availability Statement

The datasets presented in this study can be found in online repositories. The names of the repository/repositories and accession number(s) can be found below: https://www.ncbi.nlm.nih.gov/, PRJNA777819.

## Ethics Statement

The animal study was reviewed and approved by Harvard Institutional Animal Care and Use Committee.

## Author Contributions

JM, MM, SM, and KK conceived of and designed the study. KK, MM, and SM performed experiments. KK and MM analyzed and interpreted the data. MM performed transcriptomic analyses. KK and MM drafted the manuscript, which was reviewed by SM.

## Conflict of Interest

The authors declare that the research was conducted in the absence of any commercial or financial relationships that could be construed as a potential conflict of interest.

## Publisher’s Note

All claims expressed in this article are solely those of the authors and do not necessarily represent those of their affiliated organizations, or those of the publisher, the editors and the reviewers. Any product that may be evaluated in this article, or claim that may be made by its manufacturer, is not guaranteed or endorsed by the publisher.

## References

[1] Osborne TB Mendel LB Ferry EL. The effect of retardation of growth upon the breeding period and duration of life of rats. *Science.* (1917) 45:294–5. 10.1126/science.45.1160.294 17760202

[2] McCay CM Crowell MF Maynard LA. The effect of retarded growth upon the length of life span and upon the ultimate body size. 1935. *Nutrition.* (1989) 5:155–71; discussion 172. 2520283

[3] Fontana L Partridge L. Promoting health and longevity through diet: from model organisms to humans. *Cell.* (2015) 161:106–18. 10.1016/j.cell.2015.02.020 25815989 PMC4547605

[4] Greco T Glenn TC Hovda DA Prins ML. Ketogenic diet decreases oxidative stress and improves mitochondrial respiratory complex activity. *J Cereb Blood Flow Metab.* (2016) 36:1603–13. 10.1177/0271678x15610584 26661201 PMC5012517

[5] Roberts MN Wallace MA Tomilov AA Zhou Z Marcotte GR Tran D A ketogenic diet extends longevity and healthspan in adult mice. *Cell Metab.* (2017) 26:539–546 e535. 10.1016/j.cmet.2017.08.005 28877457 PMC5609489

[6] Green CL Lamming DW. Regulation of metabolic health by essential dietary amino acids. *Mech Ageing Dev.* (2019) 177:186–200. 10.1016/j.mad.2018.07.004 30044947 PMC6333505

[7] Trocha KM Kip P Tao M MacArthur MR Treviño-Villarreal JH Longchamp A Short-term preoperative protein restriction attenuates vein graft disease via induction of cystathionine γ-lyase. *Cardiovasc Res.* (2020) 116:416–28. 10.1093/cvr/cvz086 30924866 PMC8204489

[8] Solon-Biet SM Mitchell SJ Coogan SC Cogger VC Gokarn R McMahon AC Dietary protein to carbohydrate ratio and caloric restriction: comparing metabolic outcomes in mice. *Cell Rep.* (2015) 11:1529–34. 10.1016/j.celrep.2015.05.007 26027933 PMC4472496

[9] Maida A Zota A Vegiopoulos A Appak-Baskoy S Augustin HG Heikenwalder M Dietary protein dilution limits dyslipidemia in obesity through FGF21-driven fatty acid clearance. *J Nutr Biochem.* (2018) 57:189–96. 10.1016/j.jnutbio.2018.03.027 29751292

[10] MacArthur MR Mitchell SJ Treviño-Villarreal JH Grondin Y Reynolds JS Kip P Total protein, not amino acid composition, differs in plant-based versus omnivorous dietary patterns and determines metabolic health effects in mice. *Cell Metab.* (2021) 33:1808–1819.e1802. 10.1016/j.cmet.2021.06.011 34270927 PMC8478138

[11] Kennedy AR Pissios P Otu H Roberson R Xue B Asakura K A high-fat, ketogenic diet induces a unique metabolic state in mice. *Am J Physiol Endocrinol Metab.* (2007) 292:E1724–39. 10.1152/ajpendo.00717.2006 17299079

[12] Jornayvaz FR Jurczak MJ Lee HY Birkenfeld AL Frederick DW Zhang D A high-fat, ketogenic diet causes hepatic insulin resistance in mice, despite increasing energy expenditure and preventing weight gain. *Am J Physiol Endocrinol Metab.* (2010) 299:E808–15. 10.1152/ajpendo.00361.2010 20807839 PMC2980360

[13] Douris N Melman T Pecherer JM Pissios P Flier JS Cantley LC Adaptive changes in amino acid metabolism permit normal longevity in mice consuming a low-carbohydrate ketogenic diet. *Biochim Biophys Acta.* (2015) 1852(10 Pt A):2056–65. 10.1016/j.bbadis.2015.07.009 26170063 PMC4862866

[14] Bielohuby M Sisley S Sandoval D Herbach N Zengin A Fischereder M Impaired glucose tolerance in rats fed low-carbohydrate, high-fat diets. *Am J Physiol Endocrinol Metab.* (2013) 305:E1059–70. 10.1152/ajpendo.00208.2013 23982154

[15] Holland AM Kephart WC Mumford PW Mobley CB Lowery RP Shake JJ Effects of a ketogenic diet on adipose tissue, liver, and serum biomarkers in sedentary rats and rats that exercised via resisted voluntary wheel running. *Am J Physiol Regul Integr Comp Physiol.* (2016) 311:R337–51. 10.1152/ajpregu.00156.2016 27357802

[16] Grandl G Straub L Rudigier C Arnold M Wueest S Konrad D Short-term feeding of a ketogenic diet induces more severe hepatic insulin resistance than an obesogenic high-fat diet. *J Physiol.* (2018) 596:4597–609. 10.1113/JP275173 30089335 PMC6166091

[17] Park S Kim DS Kang S Daily JW III. A ketogenic diet impairs energy and glucose homeostasis by the attenuation of hypothalamic leptin signaling and hepatic insulin signaling in a rat model of non-obese type 2 diabetes. *Exp Biol Med (Maywood).* (2011) 236:194–204. 10.1258/ebm.2010.010186 21321316

[18] Treviño-Villarreal JH Reynolds JS Bartelt A Langston PK MacArthur MR Arduini A Dietary protein restriction reduces circulating VLDL triglyceride levels via CREBH-APOA5-dependent and -independent mechanisms. *JCI Insight.* (2018) 3:e99470. 10.1172/jci.insight.99470 30385734 PMC6238732

[19] Peterman MG. The ketogenic diet in epilepsy. *JAMA.* (1925) 84:1979–83.

[20] Helmholz HF. The treatment of epilepsy in childhood. *JAMA.* (1927) 88:2028–32.

[21] Talbot FB Metcalf KM Moriarty ME. A clinical study of epileptic children treated by ketogenic diet. *Boston Med Surg J.* (1927) 196:89–96. 10.1056/nejm192701201960302

[22] Barborka CJ. Ketogenic diet treatment of epilepsy in adults. *JAMA.* (1928) 91:73–8.

[23] Neal EG Chaffe H Schwartz RH Lawson MS Edwards N Fitzsimmons G The ketogenic diet for the treatment of childhood epilepsy: a randomised controlled trial. *Lancet Neurol.* (2008) 7:500–6. 10.1016/S1474-4422(08)70092-9 18456557

[24] Liu H Yang Y Wang Y Tang H Zhang F Zhang Y Ketogenic diet for treatment of intractable epilepsy in adults: a meta-analysis of observational studies. *Epilepsia Open.* (2018) 3:9–17. 10.1002/epi4.12098 29588983 PMC5839310

[25] Maschio G Oldrizzi L Tessitore N D’Angelo A Valvo E Lupo A Effects of dietary protein and phosphorus restriction on the progression of early renal failure. *Kidney Int.* (1982) 22:371–6. 10.1038/ki.1982.184 7176336

[26] Ihle BU Becker GJ Whitworth JA Charlwood RA Kincaid-Smith PS. The effect of protein restriction on the progression of renal insufficiency. *N Engl J Med.* (1989) 321:1773–7. 10.1056/NEJM198912283212601 2512486

[27] Malvy D Maingourd C Pengloan J Bagros P Nivet H. Effects of severe protein restriction with ketoanalogues in advanced renal failure. *J Am Coll Nutr.* (1999) 18:481–6. 10.1080/07315724.1999.10718887 10511331

[28] Cianciaruso B Pota A Pisani A Torraca S Annecchini R Lombardi P Metabolic effects of two low protein diets in chronic kidney disease stage 4-5–a randomized controlled trial. *Nephrol Dial Transplant.* (2008) 23:636–44. 10.1093/ndt/gfm576 17981885

[29] Riccio E Di Nuzzi A Pisani A. Nutritional treatment in chronic kidney disease: the concept of nephroprotection. *Clin Exp Nephrol.* (2015) 19:161–7. 10.1007/s10157-014-1041-7 25319188

[30] Bueno NB de Melo IS de Oliveira SL da Rocha Ataide T. Very-low-carbohydrate ketogenic diet v. low-fat diet for long-term weight loss: a meta-analysis of randomised controlled trials. *Br J Nutr.* (2013) 110:1178–87. 10.1017/S0007114513000548 23651522

[31] Larivière F Chiasson JL Schiffrin A Taveroff A Hoffer LJ. Effects of dietary protein restriction on glucose and insulin metabolism in normal and diabetic humans. *Metabolism.* (1994) 43:462–7. 10.1016/0026-0495(94)90077-9 8159104

[32] Linn T Geyer R Prassek S Laube H. Effect of dietary protein intake on insulin secretion and glucose metabolism in insulin-dependent diabetes mellitus. *J Clin Endocrinol Metab.* (1996) 81:3938–43. 10.1210/jcem.81.11.8923841 8923841

[33] Rigalleau V Combe C Blanchetier V Aubertin J Aparicio M Gin H. Low protein diet in uremia: effects on glucose metabolism and energy production rate. *Kidney Int.* (1997) 51:1222–7. 10.1038/ki.1997.167 9083290

[34] Linn T Santosa B Grönemeyer D Aygen S Scholz N Busch M Effect of long-term dietary protein intake on glucose metabolism in humans. *Diabetologia.* (2000) 43:1257–65. 10.1007/s001250051521 11079744

[35] Levine ME Suarez JA Brandhorst S Balasubramanian P Cheng CW Madia F Low protein intake is associated with a major reduction in IGF-1, cancer, and overall mortality in the 65 and younger but not older population. *Cell Metab.* (2014) 19:407–17. 10.1016/j.cmet.2014.02.006 24606898 PMC3988204

[36] Hopkins BD Pauli C Du X Wang DG Li X Wu D Suppression of insulin feedback enhances the efficacy of PI3K inhibitors. *Nature.* (2018) 560:499–503. 10.1038/s41586-018-0343-4 30051890 PMC6197057

[37] Lien EC Westermark AM Zhang Y Yuan C Li Z Lau AN Low glycaemic diets alter lipid metabolism to influence tumour growth. *Nature.* (2021) 599:302–7. 10.1038/s41586-021-04049-2 34671163 PMC8628459

[38] Schmidt M Pfetzer N Schwab M Strauss I Kammerer U. Effects of a ketogenic diet on the quality of life in 16 patients with advanced cancer: a pilot trial. *Nutr Metab (Lond).* (2011) 8:54. 10.1186/1743-7075-8-54 21794124 PMC3157418

[39] Fine EJ Segal-Isaacson CJ Feinman RD Herszkopf S Romano MC Tomuta N Targeting insulin inhibition as a metabolic therapy in advanced cancer: a pilot safety and feasibility dietary trial in 10 patients. *Nutrition.* (2012) 28:1028–35. 10.1016/j.nut.2012.05.001 22840388

[40] Rieger J Bahr O Maurer GD Hattingen E Franz K Brucker D ERGO: a pilot study of ketogenic diet in recurrent glioblastoma. *Int J Oncol.* (2014) 44:1843–52. 10.3892/ijo.2014.2382 24728273 PMC4063533

[41] Goldberg EL Shchukina I Asher JL Sidorov S Artyomov MN Dixit VD. Ketogenesis activates metabolically protective gammadelta T cells in visceral adipose tissue. *Nat Metab.* (2020) 2:50–61. 10.1038/s42255-019-0160-6 32694683 PMC10150608

[42] Newman JC Covarrubias AJ Zhao M Yu X Gut P Ng CP Ketogenic diet reduces midlife mortality and improves memory in aging mice. *Cell Metab.* (2017) 26:547–557 e548. 10.1016/j.cmet.2017.08.004 28877458 PMC5605815

[43] de Groot S Lugtenberg RT Cohen D Welters MJP Ehsan I Vreeswijk MPG Fasting mimicking diet as an adjunct to neoadjuvant chemotherapy for breast cancer in the multicentre randomized phase 2 DIRECT trial. *Nat Commun.* (2020) 11:3083. 10.1038/s41467-020-16138-3 32576828 PMC7311547

[44] Brandhorst S. Fasting and fasting-mimicking diets for chemotherapy augmentation. *Geroscience.* (2021) 43:1201–16. 10.1007/s11357-020-00317-7 33410090 PMC8190229

[45] Wei M Brandhorst S Shelehchi M Mirzaei H Cheng CW Budniak J Fasting-mimicking diet and markers/risk factors for aging, diabetes, cancer, and cardiovascular disease. *Sci Transl Med.* (2017) 9:eaai8700. 10.1126/scitranslmed.aai8700 28202779 PMC6816332

[46] Brandhorst S Choi IY Wei M Cheng CW Sedrakyan S Navarrete G A periodic diet that mimics fasting promotes multi-system regeneration, enhanced cognitive performance, and healthspan. *Cell Metab.* (2015) 22:86–99. 10.1016/j.cmet.2015.05.012 26094889 PMC4509734

[47] Robertson LT Trevino-Villarreal JH Mejia P Grondin Y Harputlugil E Hine C Protein and calorie restriction contribute additively to protection from renal ischemia reperfusion injury partly via leptin reduction in male mice. *J Nutr.* (2015) 145:1717–27. 10.3945/jn.114.199380 26041674 PMC4516761

[48] Jongbloed F de Bruin RWF Klaassen RA Beekhof P van Steeg H Dor FJMF Short-term preoperative calorie and protein restriction is feasible in healthy kidney donors and morbidly obese patients scheduled for surgery. *Nutrients.* (2016) 8:306. 10.3390/nu8050306 27213441 PMC4882718

[49] Kip P Trocha KM Tao M O’Leary JJ Ruske J Giulietti JM Insights from a short-term protein-calorie restriction exploratory trial in elective carotid endarterectomy patients. *Vasc Endovascular Surg.* (2019) 53:470–6. 10.1177/1538574419856453 31216949

[50] Rothwell NJ Stock MJ Tyzbir RS. Mechanisms of thermogenesis induced by low protein diets. *Metabolism.* (1983) 32:257–61. 10.1016/0026-0495(83)90190-7 6827996

[51] Fisher FM Maratos-Flier E. Understanding the physiology of FGF21. *Annu Rev Physiol.* (2016) 78:223–41. 10.1146/annurev-physiol-021115-105339 26654352

[52] Hill CM Laeger T Albarado DC McDougal DH Berthoud HR Munzberg H Low protein-induced increases in FGF21 drive UCP1-dependent metabolic but not thermoregulatory endpoints. *Sci Rep.* (2017) 7:8209. 10.1038/s41598-017-07498-w 28811495 PMC5557875

[53] Geng L Lam KSL Xu A. The therapeutic potential of FGF21 in metabolic diseases: from bench to clinic. *Nat Rev Endocrinol.* (2020) 16:654–67. 10.1038/s41574-020-0386-0 32764725

[54] Paoli A Rubini A Volek JS Grimaldi KA. Beyond weight loss: a review of the therapeutic uses of very-low-carbohydrate (ketogenic) diets. *Eur J Clin Nutr.* (2013) 67:789–96. 10.1038/ejcn.2013.116 23801097 PMC3826507

[55] Yu G Wang LG Han Y He QY. clusterProfiler: an R package for comparing biological themes among gene clusters. *OMICS.* (2012) 16:284–7. 10.1089/omi.2011.0118 22455463 PMC3339379

[56] Langfelder P Horvath S. WGCNA: an R package for weighted correlation network analysis. *BMC Bioinformatics.* (2008) 9:559. 10.1186/1471-2105-9-559 19114008 PMC2631488

[57] Gearing LJ Cumming HE Chapman R Finkel AM Woodhouse IB Luu K CiiiDER: a tool for predicting and analysing transcription factor binding sites. *PLoS One.* (2019) 14:e0215495. 10.1371/journal.pone.0215495 31483836 PMC6726224

[58] Lopez-Dominguez JA Ramsey JJ Tran D Imai DM Koehne A Laing ST The influence of dietary fat source on life span in calorie restricted mice. *J Gerontol A Biol Sci Med Sci.* (2015) 70:1181–8. 10.1093/gerona/glu177 25313149 PMC4612357

[59] El Akoum S Lamontagne V Cloutier I Tanguay JF. Nature of fatty acids in high fat diets differentially delineates obesity-linked metabolic syndrome components in male and female C57BL/6J mice. *Diabetol Metab Syndr.* (2011) 3:34. 10.1186/1758-5996-3-34 22166251 PMC3277487

[60] Buettner R Parhofer KG Woenckhaus M Wrede CE Kunz-Schughart LA Scholmerich J Defining high-fat-diet rat models: metabolic and molecular effects of different fat types. *J Mol Endocrinol.* (2006) 36:485–501. 10.1677/jme.1.01909 16720718

[61] Badman MK Kennedy AR Adams AC Pissios P Maratos-Flier E. A very low carbohydrate ketogenic diet improves glucose tolerance in ob/ob mice independently of weight loss. *Am J Physiol Endocrinol Metab.* (2009) 297:E1197–204. 10.1152/ajpendo.00357.2009 19738035 PMC2781352

[62] Sherrier M Li H. The impact of keto-adaptation on exercise performance and the role of metabolic-regulating cytokines. *Am J Clin Nutr.* (2019) 110:562–73. 10.1093/ajcn/nqz145 31347659

[63] Badman MK Koester A Flier JS Kharitonenkov A Maratos-Flier E. Fibroblast growth factor 21-deficient mice demonstrate impaired adaptation to ketosis. *Endocrinology.* (2009) 150:4931–40. 10.1210/en.2009-0532 19819944 PMC2775979

[64] Stemmer K Zani F Habegger KM Neff C Kotzbeck P Bauer M FGF21 is not required for glucose homeostasis, ketosis or tumour suppression associated with ketogenic diets in mice. *Diabetologia.* (2015) 58:2414–23. 10.1007/s00125-015-3668-7 26099854 PMC5144740

[65] Laeger T Henagan TM Albarado DC Redman LM Bray GA Noland RC FGF21 is an endocrine signal of protein restriction. *J Clin Invest.* (2014) 124:3913–22. 10.1172/JCI74915 25133427 PMC4153701

[66] Solon-Biet SM Cogger VC Pulpitel T Heblinski M Wahl D McMahon AC Defining the nutritional and metabolic context of FGF21 using the geometric framework. *Cell Metab.* (2016) 24:555–65. 10.1016/j.cmet.2016.09.001 27693377

[67] Hill CM Laeger T Dehner M Albarado DC Clarke B Wanders D FGF21 signals protein status to the brain and adaptively regulates food choice and metabolism. *Cell Rep.* (2019) 27:2934–2947.e2933. 10.1016/j.celrep.2019.05.022 31167139 PMC6579533

[68] Cota D Proulx K Smith KA Kozma SC Thomas G Woods SC Hypothalamic mTOR signaling regulates food intake. *Science.* (2006) 312:927–30. 10.1126/science.1124147 16690869

[69] Wu Y Li B Li L Mitchell SE Green CL D’Agostino G Very-low-protein diets lead to reduced food intake and weight loss, linked to inhibition of hypothalamic mTOR signaling, in mice. *Cell Metab.* (2021) 33:888–904.e886. 10.1016/j.cmet.2021.01.017 33667386

[70] Fisher FM Chui PC Antonellis PJ Bina HA Kharitonenkov A Flier JS Obesity is a fibroblast growth factor 21 (FGF21)-resistant state. *Diabetes.* (2010) 59:2781–9. 10.2337/db10-0193 20682689 PMC2963536

