# Supplementary material for: Short-Term Ketogenic Diet Induces a Molecular Response That Is Distinct From Dietary Protein Restriction
Source: Front Nutr. 2022 Mar 30;9:839341. doi: 10.3389/fnut.2022.839341 (PMC9005751; doi:10.3389/fnut.2022.839341)
Supplement: Supplementary file 1 [file Data_Sheet_1.PDF]

Supplementary Material

1 Supplementary Figures

A

Diet	% Fat	% Carbs	% Protein	Energy (kcal/g)
CTL	10	70	20	1.9
PF	10	90	0	1.9
KD0P	90	10	0	3.0
KD5P	90	5	5	3.0
KD10P	90	0	10	3.0
KD20P	80	0	20	2.7

B

Diet	D12450BSpx	D10070801Lpx	Cocoa Butter	Casein	Sucrose	Water	Agar
CTL	205	0	0	45	0	250	5
PF	205	0	0	0	45	250	5
KD0P	0	51.5	158.8	0	39.7	281.3	5.6
KD5P	0	51.5	158.8	19.9	19.9	281.7	5.6
KD10P	0	51.5	158.8	39.7	0	282.1	5.6
KD20P	0	51.5	127	71.5	0	282.1	5.6

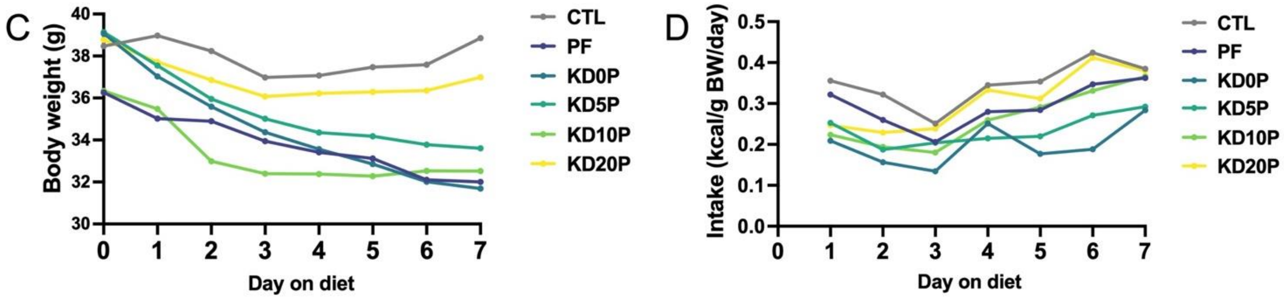

Supplementary Figure 1. (A) Table of macronutrient compositions and energy densities of the experimental diets. **(B)** Table of ingredients in experimental diets in grams. **(C)** Body weights in grams across 7 days of feeding experiments diets. **(D)** Energy intakes in kcals per gram body weight per day across 7 days of feeding experimental diets. Intake was normalized to the summed body weight of 2 mice co-housed per cage and presented per cage.

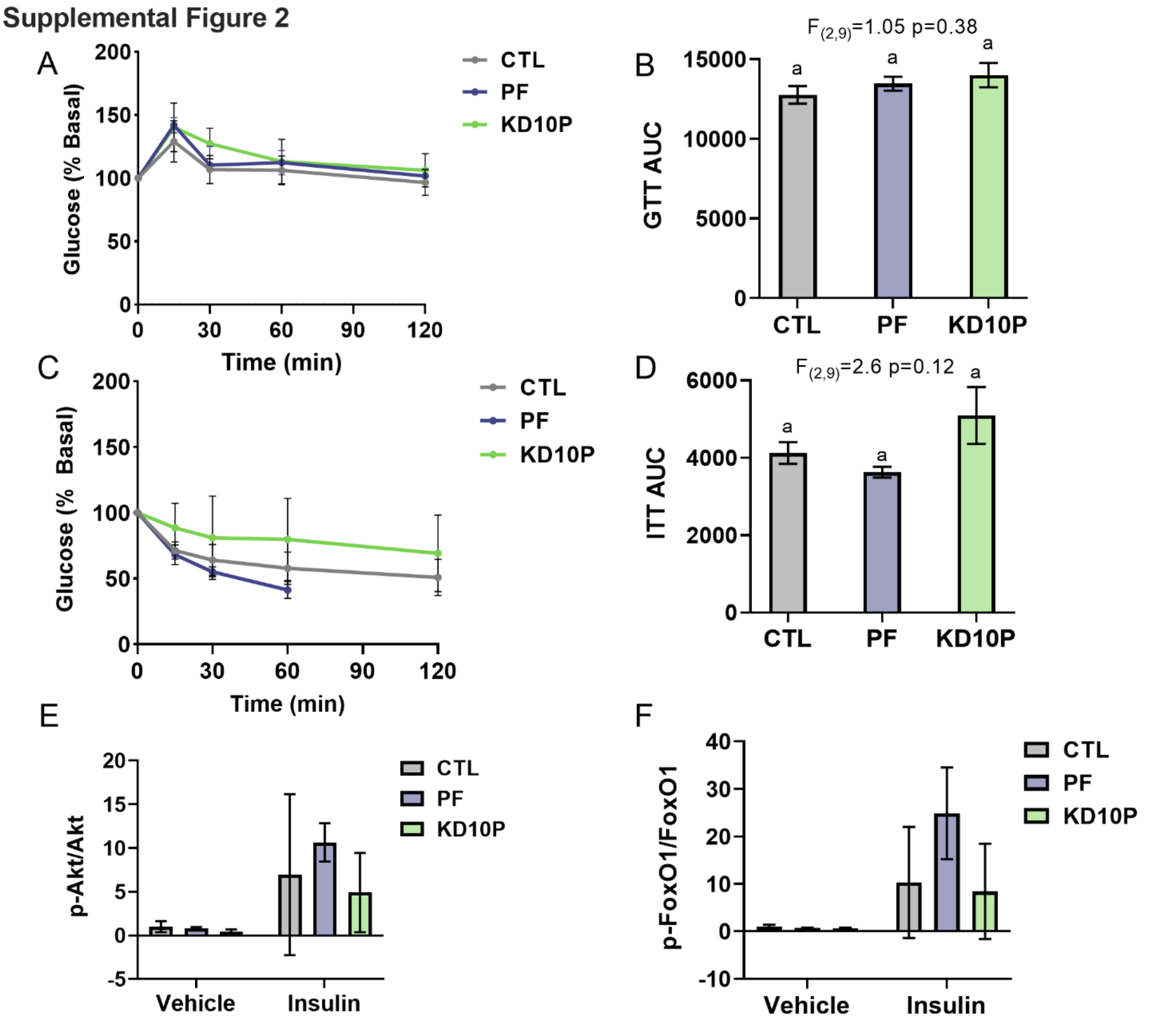

Supplementary Figure 2. (A) Blood glucose levels during oral glucose tolerance test, normalized to baseline and **(B)** corresponding area under the curve (AUC). **(C)** Blood glucose levels during insulin tolerance test normalized to baseline and **(D)** corresponding AUC. **(E)** Quantification of phospho-AKT normalized to total AKT by western blot. **(F)** Quantification of phospho-FoxO1 normalized to total FoxO1 by western blot.

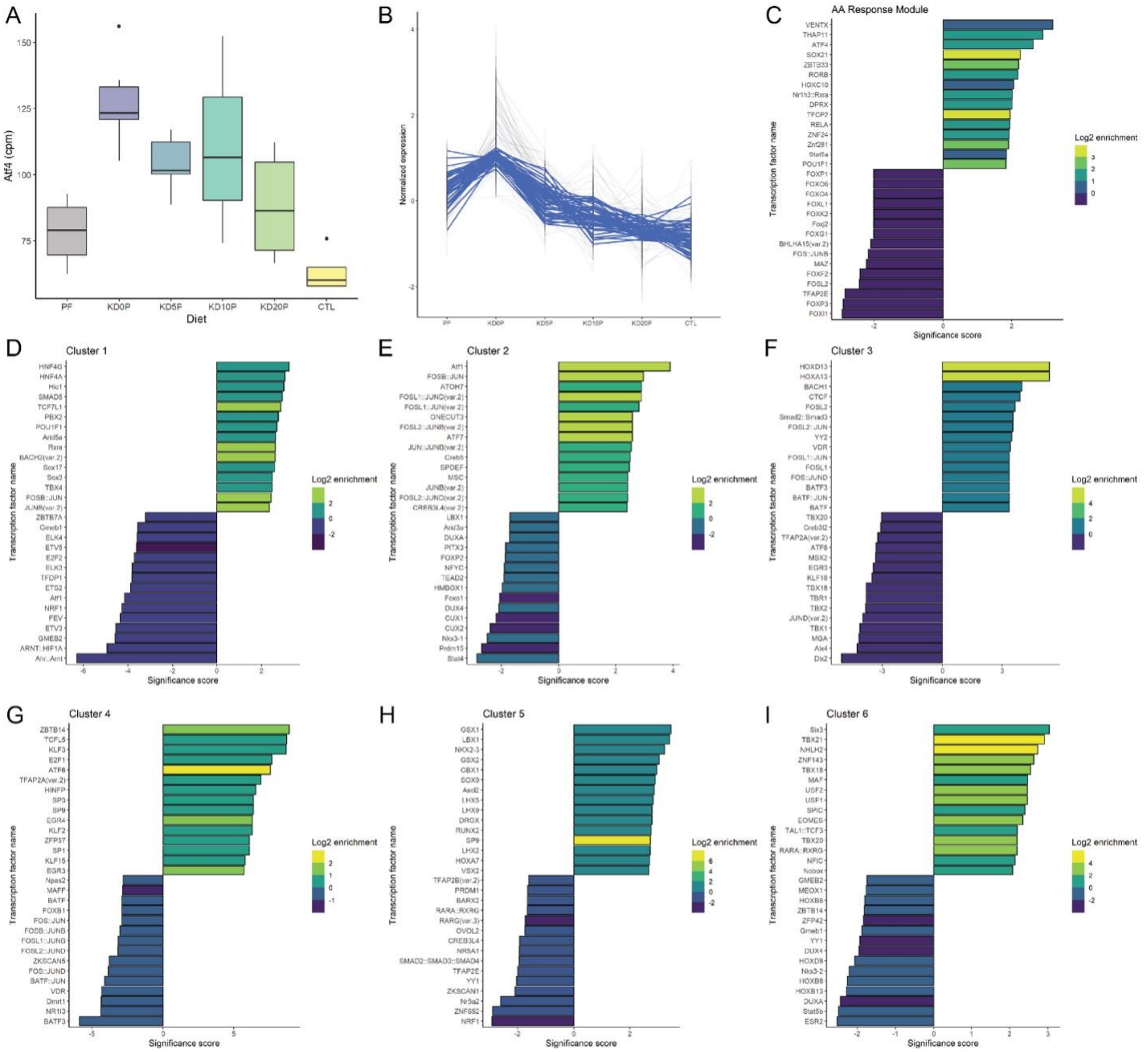

Supplementary Figure 3. (A) Hepatic Atf4 transcript levels as counts per million (cpm) across experimental diets. **(B)** Weighted gene correlation network analysis module containing amino acid responsive genes including Psat1, Asns and Fgf21. **(C)** Top 10 enriched and depleted transcription factor binding motifs corresponding to the module in B. **(D-I)** Top 10 enriched and depleted transcription factor binding motifs in clusters 1 through 6.

D12450BS and D12450BSpx

Water Suspendible Rodent Diet With 10 kcal% Fat
and Same without Added Protein

Product #	D12450BS		D12450BSpx	
%	gm	kcal	gm	kcal
Protein	19.2	20.0	0.0	0.0
Carbohydrate	67.3	70.0	83.3	87.5
Fat	4.3	10.0	5.3	12.5
Total		100.0		100.0
kcal/gm	3.85		3.81	
**Ingredient**	**gm**	**kcal**	**gm**	**kcal**
Casein, 80 Mesh	0	0	0	0
Casein, Enzyme Hydrolyzed	200	800	0	0
L-Cystine	3	12	0	0
Sucrose	350	1400	350	1400
Maltodextrin 42	350	1400	350	1400
Cellulose, BW200	40	0	40	0
Xanthan Gum	10	0	10	0
Soybean Oil	25	225	25	225
Lard	20	180	20	180
Mineral Mix S10011	0	0	0	0
Mineral Mix S10026	10	0	10	0
DiCalcium Phosphate	13	0	13	0
Calcium Carbonate	5.5	0	5.5	0
Potassium Citrate, 1 H2O	16.5	0	16.5	0
Vitamin Mix V10001	10	40	10	40
Choline Bitartrate	2	0	2	0
FD&C Yellow Dye #5	0.05	0	0.05	0
**Total**	**1055.05**	**4057**	**852.05**	**3245**

Table S1 Composition of low fat base diet

D10070801L And D10070801Lpx

Formulated by:
Research Diets, Inc.
Steven Yeung
December 2018

Water Suspendible Rodent Diet With 90 kcal% Fat (Mostly Cocoa Butter)

Product #	D10070801			D10070801L		D10070801Lpx	
	gm%	kcal%		gm%	kcal%	gm%	kcal%
Protein	17	10		17	10	0	0
Carbohydrate	0	0		0	0	0	0
Fat	67	90		67	90	20	100
Total		100			100		100
kcal/gm	6.7			6.7		1.8	
**Ingredient**	**gm**	**kcal**		**gm**	**kcal**	**gm**	**kcal**
Casein	100	400		100	400	0	0
L-Cystine	1.5	6		1.5	6	0	0
Corn Starch	0	0		0	0	0	0
Maltodextrin 10	0	0		0	0	0	0
Sucrose	0	0		0	0	0	0
Cellulose, BW200	50	0		40	0	40	0
Xanthan Gum	0	0		10	0	10	0
Soybean Oil	25	225		25	225	25	225
Lard	0	0		0	0	0	0
Cocoa Butter	381	3429		381	3429	0	0
Primex	0	0		0	0	0	0
Mineral Mix, S10026	10	0		10	0	10	0
DiCalcium Phosphate	13	0		13	0	13	0
Calcium Carbonate	5.5	0		5.5	0	5.5	0
Potassium Citrate, 1 H2O	16.5	0		16.5	0	16.5	0
Vitamin Mix, V10001	0	0		0	0	0	0
Vitamin Mix, V10001C,10x vitamins	1	0		1	0	1	0
Choline Bitartrate	2	0		2	0	2	0
Cholesterol	0	0		0	0	0	0
Sodium Cholic Acid	0	0		0	0	0	0
FD&C Yellow Dye #5	0.025	0		0.025	0	0.025	0
FD&C Red Dye #40	0.025	0		0.025	0	0.025	0
FD&C Blue Dye #1	0	0		0	0	0	0
**Total**	**605.55**	**4060**		**605.55**	**4060**	**123.05**	**225**

Research Diets, Inc.
20 Jules Lane
New Brunswick, NJ 08901 USA
info@researchdiets.com

MacArthurM02.for

www.ResearchDiets.com

Table S2 Composition of high fat base diet
